# Structural and Electrical Characterization of LaSrAl_1−x_Mg_x_O_4−δ_ Layered Perovskites Obtained by Mechanical Synthesis

**DOI:** 10.3390/ma16247564

**Published:** 2023-12-08

**Authors:** Carlos Mariño, Daniel Serafini, Juan Basbus, José Antonio Alonso, Loreto Troncoso

**Affiliations:** 1 Departamento de Física, Universidad de Santiago de Chile, Avenida Libertador Bernardo O’Higgins nº 3363, Estación Central, Santiago 9170124, Chile; daniel.serafini@usach.cl; 2 Department of Civil, Chemical and Environmental Engineering (DICCA), University of Genova (UniGe), Via all’Opera Pia 15, 16145 Genoa, GE, Italy; juanbasbus@hotmail.com; 3 Instituto de Ciencia de Materiales de Madrid (ICMM), CSIC, Cantoblanco, 28049 Madrid, Spain; 4 Instituto de Ingeniería Mecánica, Universidad austral de Chile, General Lagos 2086, Valdivia 5111187, Chile; 5 MIGA Millennium Institute (ICN2021_023), Santiago 8320211, Chile

**Keywords:** crystallographic properties, mechano-chemical synthesis, electron diffraction, ionic conductivity, layer perovskites, SOFC electrolytes

## Abstract

This work presents the structural and electrical characterization of K_2_NiF_4_-type layered perovskites of LaSrAl_1−x_Mg_x_O_4−δ_ composition to be used as oxide–ion electrolytes for a solid-oxide fuel cell (SOFC). These perovskites were prepared by mechano-chemical synthesis (ball milling), an alternative to traditional synthesis methods such as citrate-nitrates and solid-state reaction. With these methods, two things are avoided: first, the use of nitrate salts, which are more environmentally harmful than oxide precursors, and second, it saves the series of long thermal treatments associated with solid-state reactions. After grinding the precursors, nanometric particles were obtained with a combination of crystalline regions and amorphous regions; this effect was determined by XRD and TEM, showing that Mg has a positive effect on the phase formation by only mechanical synthesis. R2C4: After sintering, it was found by XRD that the sample x = 0.1 only presents the diffraction peaks corresponding to the desired phase, which shows a phase purity greater than 97%, even higher than that of the standard undoped sample. For x = 0.2 and 0.3, there was a segregation of impurities, possibly by the local migration of La and Sr heavy cations; this was determined by SEM and EDS. The electrical characterization of the sintered pellets was carried out by electrochemical impedance spectroscopy, which determined that the incorporation of Mg in the structure improves the ionic conductivity by three orders of magnitude, obtaining conductivities of 1.6 mS/cm at 900 °C for x = 0.2. Although the improvement in conductivity is considerable, many challenges such as densification, the segregation of impurities, and the study of mechanical and thermal properties must be carried out on these materials to endorse them as solid electrolytes in SOFC.

## 1. Introduction

The effects of climate change are becoming progressively severe and, in the last years, have turn out to be a real threat. CO_2_ emissions due to energy use correspond to 76% of global releases, triggering unwanted mechanisms such as global warming, weather instability, uncontrolled floods, etc. For this reason, the implementation of clean technologies urgently needs to be set up [1,2].

One of the fundamental technologies driving the decarbonization of the energy system is the use of fuel cells, in combination with clean fuels like hydrogen, particularly Solid Oxide Fuel Cell (SOFC) [3,4]. Its development deploys multidisciplinary teams that encompass the study of physical-chemical properties of complex ceramics, the improvement of computational models, as well as materials science and engineering. The joint development of all these lines of research points to electrochemical devices designed to produce electricity (fuel cells) or to generate green hydrogen through primary sources (electrolyzers) [5]. Faced with the diversity of designs and operating temperatures, solid-oxide fuel cells and solid-oxide electrolyzers are the most promising candidates due to their cogeneration capacity (SOFC-HCP) [6], their high operating power, and their industrial scalability.

One of the common components of both solid oxide fuel cells and electrolyzers is the solid oxide electrolyte. It consists of a crystalline material that allows for the transport of oxygen ions across the crystalline structure. The journey of oxygen begins at the cathode material where O_2_ from the air is reduced to its anionic spice (O^2−^), which is transferred from the crystal lattice of the porous cathode to the crystal lattice of the dense electrolyte. Here, it is preferentially transported by thermally activated mechanisms, finally jumping from the crystal lattice of the electrolyte to the crystal lattice of the porous anode, where it oxidizes the fuel used for the electrochemical reaction. The versatility of the systems includes fuels such as pure hydrogen, methane, carbon monoxide, ammonia [7] and others. The movement of the ions can be characterized as an ionic current; the resistance of the material can be measured by reliable methods such as electrochemical impedance spectroscopy at high temperatures (EISHT) or conductivity under the partial pressure of oxygen (PPOC). A wide variety of solid electrolytes with their respective crystal structures are available for the diverse range of electrodes already established in the market, as well as novel materials experimentally tested at the laboratory level. The electrolyte of excellence is YSZ (yttria-stabilized zirconia) at high temperatures and GDC (gadolinium-doped ceria) or LSGM (lanthanum–strontium–gallium–magnesium oxide) at intermediate temperatures [8,9,10]. These complex ceramics are generally obtained by solid state reactions. 

There are alternative crystalline structures such as K_2_NiF_4_-type layered perovskites, which have attracted the attention of researchers for their non-stoichiometric capabilities, enabling the incorporation of anionic vacancies and/or interstitial oxygen atoms, which greatly improve their electrical properties [11]. In previous works, we synthesized K_2_NiF_4_-type perovskites with LaSrAl_1−x_Mg_x_O_4−δ_ [12] and LaSrGa_1−x_Mg_x_O_4−δ_ [13] compositions, where the incorporation of divalent Mg to the parent LaSrAlO_4_ or LaSrGaO_4_ oxides improved the conductivity by three orders of magnitude. Crystallographic studies by neutron diffraction allowed us to characterize the oxygen diffusional pathways, showing that the open structure favors the movement of the oxygen ions across the crystal through the interstitial sites of the structure, thus obtaining conductivities up to 3 mS/cm at 900 °C [12,13].

The most used methods for the development of these materials are citrate-nitrate synthesis followed by solid-state reactions. In this context, the reagent costs of citrate-nitrate routes are higher than those of oxides; meanwhile, the higher sintering temperatures required for solid-state reactions involved higher energy costs than those involved by wet chemistry techniques. Mechano-chemical synthesis is an alternative method that saves preliminary solid-state-reaction steps. This method consists of the mechanical milling of precursor oxides in the solid state, within a high or medium energy ball mill, followed by a final annealing or direct sintering thermal treatment. This process produces homogeneous powders with a high density of surface defects, which improves sinterability properties and decreases sintering temperatures [14]. Another benefit is that the milling conditions allow for obtaining nanocrystalline particles of the desired phase and, at the same time, act as nucleation seeds for sintering [15]. This work describes the preparation of selected oxides of the series of layered perovskites of the composition LaSrAl_1−x_Mg_x_O_4_, followed by a complete characterization by the pertinent techniques, as described below.

## 2. Experimental Methodology

The departure reactant oxides were La_2_O_3_ (99.95% purity), SrO (99.95% purity), Al_2_O_3_ (99.95% purity) and MgO (99.95% purity), all Sigma Aldrich (St. Louis, MO, USA). They were annealed at 1000 °C for 4 h to remove metal carbonates and eliminate the hydration of the precursors; immediately after annealing, they were weighed on a high precision balance (0.0001 g) to obtain the desired stoichiometry. The mixture was placed in a container coated with tungsten carbide together with 40 balls of a 5 mm diameter of the same material. A ball:powder ratio of 10:1 was used, and mechano-synthesis was carried out using a mill (Micro-Mill Pulverisette model 7, Fritsch, Idar-Oberstein, Germany) at a speed of 600 rpm for 10 h at 10 min grinding and resting intervals.

Phase formation was checked by X-ray diffraction (XRD) with a Bruker D2Phaser diffractometer (Billerica, MA, USA). This instrument has Bragg–Brentano geometry, Cu Kα radiation, 30 kV and 10 mA at 0.02°/min between 20 and 80°. The diffractograms were analyzed by the Rietveld method using FU LLPROF [16]. To support the crystallinity study, the powders were analyzed by TEM PHILLIPS 300 equipment (Amsterdam, The Netherlands).

The thermometric study was performed with differential scanning calorimetry and thermogravimetry (DSC-TG) using a TA Discovery SDT650 model, (New Castle, DE, USA). The powders were cycled between room temperature and 1200 °C in air at a heating rate of 20 °C/min until phase formation. Subsequently, the powders were uniaxially compacted at 50 kg/cm^2^ and sintered at 1400 °C for 12 h.

The morphology and microstructure of the dense pellets were analyzed by Scanning Electron Microscopy using an Inspect S50 FEI microscope, (Midland, ON, Canada) equipped with Energy Dispersive Spectroscopy (EDS) detectors for elemental analysis, and then the pellets were included in a chamber (using Struers CitoVac, Champigny, France); to obtain the specular surface, the samples were polished using 0.5 μm silica wipes.

The porosity was studied using backscattered electrons and calculated by contrast analysis using Image J. The electrical properties of the materials were studied by electrochemical impedance spectroscopy (EIS). The samples were sintered at 1400 °C for 10 h in air to ensure good densification. Impedance spectra were measured on disk geometries painted on both sides with Pt ink and a platinum current collector. A tube furnace was used to anneal the samples. An Autolab PGSTAT30, (Herisau, Switzerland) 1 MHz to 1 mHz potentiostat/galvanostat with a 50 mV amplitude between 300 and 900 °C in air was used to perform EIS measurements. The spectra were fitted with Equivalent Electrical Circuits (EEC), using the EIS Spectrum Analyzer [17].

## 3. Results and Discussion

### 3.1. Structural Characterization

**Figure 1:**
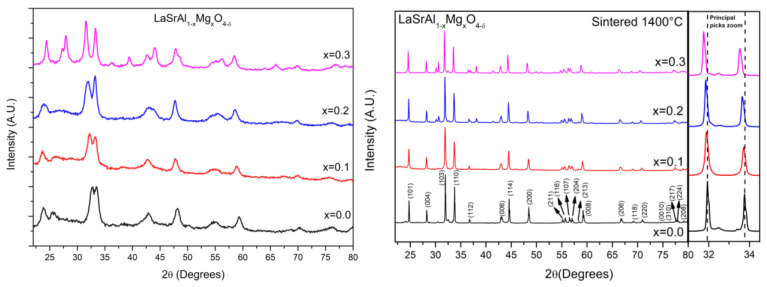
X-ray diffraction (XRD) patterns collected with Cu Kα radiation. (**Left**): XRD patterns of powders ground for 100 h for the parent LaSrALO_4_ oxide and those of the Mg-doped samples LaSrAl_1−x_Mg_x_O_4−δ_ (x = 0.1–0.3). An improvement in the crystallinity is observed as Mg is incorporated. (**Right**): XRD patterns of_,_ the sintered samples at 1400 °C of the parent and doped samples; the zoom of the graph shows the main peaks of the (103) and (110) reflections.

**Figure 2:**
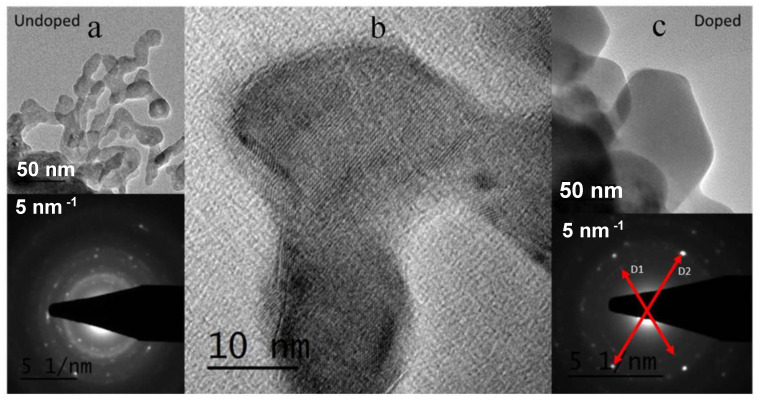
TEM images and electron diffraction patterns of the parent LaSrAlO_4_ and Mg-doped LaSrAl_0.7_Mg_0.3_O_4−δ_ samples. (**a**): LaSrAlO_4_ nanoparticles and electron diffraction pattern. (**b**): Nanoparticle micrograph of the LaSrAlO_4_ sample. (**c**): LaSrAl_0.7_Mg_0.3_O_4−δ_ nanoparticles and their electron diffraction pattern, D1(hkl) = 101 D2(hkl) = 103 planes.

**Figure 3:**
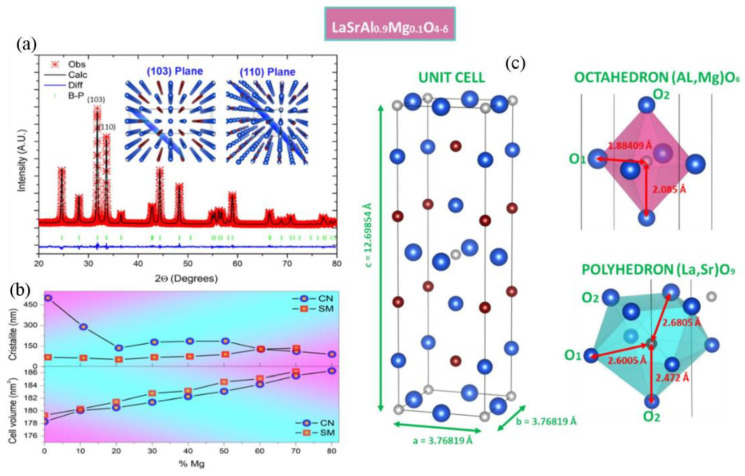
Structural characterization: (**a**) X-ray diffractogram measured and calculated after Rietveld analysis of the single phase of LaSrAl_0.9_Mg_0.1_O_4−δ_. The crosses (×) correspond to the measured pattern and the black line (-) corresponds to the calculated pattern; the blue line is the difference (-) and the bars (|) correspond to the Bragg diffraction peaks. The onset is a graphic representation of the principal planes (103) and (110). (**b**) Crystallite size and unit-cell volume obtained by mechanical synthesis and chemical synthesis (previous work [12]). (**c**) Visualization of the unit cell of the layered perovskite (K_2_NiF_4_ type) of LaSrAl_0.7_Mg_0.3_O_4−−δ_ (x = 0.1–0.3) and its respective coordination polyhedra. The interatomic distances between cations and anions are also included.

X-ray diffractograms of the magnesium-doped lanthanum aluminate powders obtained after mechano-synthesis are shown in Figure 1. The ball milling process allowed for the formation of the expected phase with broad peaks, revealing a particle size of a nanometer dimension. The incorporation of Mg in the synthesis allows for the better crystallization of the product, involving a better resolution of the diffraction peaks; this fact suggests that the presence of Mg promotes the formation and crystal growth of the K_2_NiF_4_-type phase. TEM transmission electron microscopy was performed to obtain information about the microstructure of the mechanically synthesized powders. Figure 2 shows an image of the sample doped with 30% Mg. The size of the particles is of the order of 10 nm in diameter. The electron diffraction image shows crystallinity which complements the X-ray diffraction observations. A diffuse background corresponding to an amorphous zone of the material belonging to an incompletely transformed phase is also observed. After mechanical synthesis, the powders were annealed at 1100 °C and then sintered at 1400 °C. The pellets were characterized by XRD, and the diffractograms are shown in Figure 3. For x = 0.0 (LaSrAlO_4_), the obtained phase is well defined in the space group I4/mmm. A good crystallization of the material is observed, and all Miller indexes of the crystal structure are identified. It is seen that the standard sample presents LaSrAlO_4_ minor impurities between the main peaks (103) and (220). For the Mg-doped samples, a shift of the diffraction peaks is observed; the zoom of the main peaks is displayed on the right of the diffractograms. The shift is mainly due to the presence of Mg^2+^ in the crystallographic site of Al^3+^; the larger ionic size of the former, by 25%, produces an increase in the unit cell volume. The crystal structures were also refined from XRD data using the Rietveld method, which allows for a complete characterization of lattice parameters, atomic positions, etc. Figure 3 illustrates the refinements for sample x = 0.1, which is completely single-phase. This was performed for the samples annealed at 1100 °C. Figure 3a shows the diffraction pattern obtained for the LaSrAl_0.9_Mg_0.1_O_4−δ_ sample. The model used for the refinement was a tetragonal structure of the type K_2_NiF_4_ of the space group I4/mmm (# 139). The calculated crystallographic parameters are shown in Table 1. The information in this table reveals that, as the Mg content increases, the size of the lattice parameters also increases, particularly for the c-axis of the unit cell, due to the larger size of Mg^2+^ ions, as commented above. The isotropic thermal factors obtained show that the O1 oxygens have a similar degree of freedom as the O2 oxygens. The occupancy factor for both oxygen atoms could not be refined from laboratory XRD data; thus, they were fixed to the stoichiometric value. However, it must be considered that the incorporation of Mg into the LaSrAlO_4_ lattice forcedly implies the creation of oxygen vacancies, driven by the substitution of Mg^2+^ by Al^3+^ ions. A neutron diffraction study would be required for the quantification of such oxygen vacancies.

The main diffraction planes (103) and (110) are observed in the inset of Figure 3a. The plot in Figure 3b shows the results of the unit-cell volumes and crystallite size (coherent diffraction pattern) of the Mg series obtained by SM mechanical synthesis (this work) and CN citrate–nitrate chemical synthesis (previous work) [12], both annealed at 1100 °C. It is seen that the unit-cell volume is slightly larger by SM, which may reveal a better tendency to incorporate Mg at the 2*a* (1/2 1/2 1/2) site of Al. The crystallite size for the SM is also lower than that of the crystallites obtained by CN synthesis. This makes them interesting in exploring fast sintering techniques.

From the refinements, the interatomic distances shown in Table 2 were also obtained. Figure 3c illustrates these distances using VESTA software (4.6.0). It is observed that the oxygens have a greater distance to their central cation in the polyhedron [La,Sr]O_9_ versus the octahedron [AlMg]O_6_. Therefore, the coordination has a more ionic character for the A-type cations and a more covalent character for the B-type cations.

### 3.2. Thermal Stability

**Figure 4:**
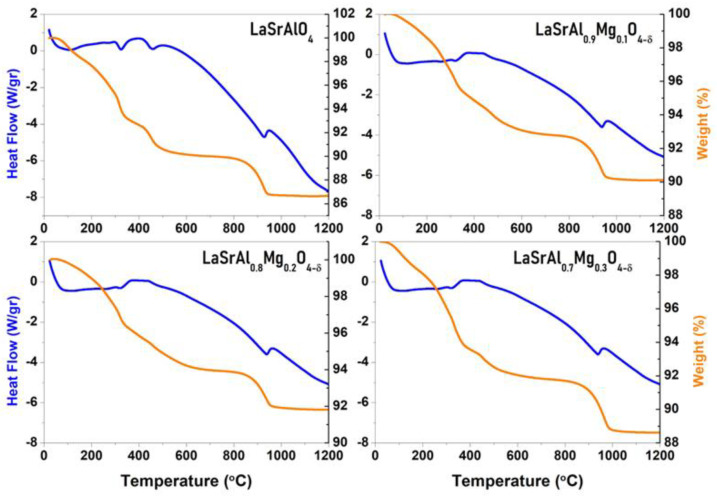
Differential scanning calorimetry (left axes) and thermogravimetry curves (right axes) of K_2_NiF4-type perovskites for LaSrAl_1−x_Mg_x_O_4−δ_ (x = 0.0–0.3).

The results obtained in the DSC and TG study are shown in Figure 4. The orange-colored curves (left axes) show the heat flux per unit mass and reveal an endothermic peak at around 900 °C. The size of the peak is smaller than that obtained by chemical synthesis in a previous work [13]. The peak thus corresponds to the phase crystallization from the amorphous component, not transformed by mechanical synthesis. This complements the studies obtained by X-ray diffraction and transmission electron microscopy, which revealed that the desired phase is obtained mechanically without annealing treatment. It is also observed that the incorporation of Mg has a small effect of 20 °C on the transformation temperature. All samples experienced a mass loss in the order of 10%. The first 8% of mass is lost in the range between 100 and 650 °C. This is mainly due to the strong hydration that the samples underwent prior to the analysis and also the decomposition of strontium carbonates that may have formed from the untransformed strontium oxide; this had already been noticed by Zvareva and Popova et al., who made a complete study of the transformations undergone by perovskites of the Ruddlesden Popper series [18,19]. The last 2% of mass loss is due to the crystallization of the phase from the incompletely transformed phase.

### 3.3. Microstructure

**Figure 5:**
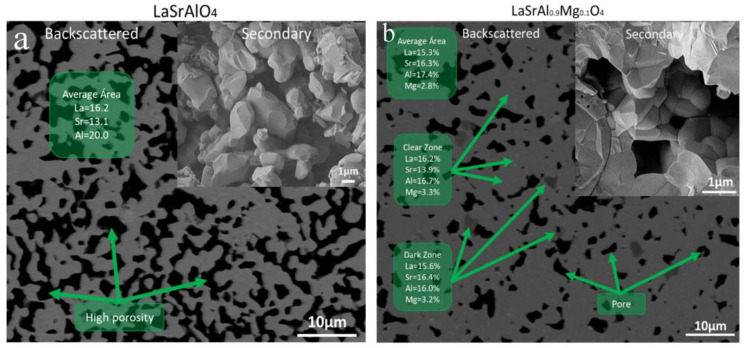
Scanning electron microscopy images of K_2_NiF_4_-type perovkites with an LaSrAl_1−x_Mg_x_O_4−δ_ (x = 0.0–0.1) composition after sintering. The green boxes present the atomic percentage data obtained by X-ray dispersive spectroscopy (EDS). (**a**) Backscattered electron and (**b**) secondary electron micrographs are presented.

The microstructure has been examined by scanning electron microscopy and X-ray scattering analysis. Figure 5 shows the micrograph obtained from the parent sample (x = 0.0) and the Mg-doped oxide with x = 0.1. The micrographs are presented using back-scattered electrons and secondary electrons. In Figure 5a, the gray region corresponds to LaSrAlO_4_, and no compositional segregations are observed. The black region corresponds to the pores; using Image J, the area ratio was calculated, and a densification of the order of 70% was obtained. The secondary electron image shows rounded grains where the pellets were broken and a grain size in the order of 1 µm, smaller than that of a traditional ceramic of about 10 µm [20]. Figure 5b is the micrograph for x = 0.1. The backscattered electron image shows that Mg-doped LaSrAlO_4_ has minimal segregation in the dark gray regions where there is a higher Sr content at an La/Sr = 0.95 ratio. The light gray area shows an La/Sr = 1.16 ratio, which does not generate a great impact on Mg segregation, as seen in previous work with chemical synthesis [12]. The black region corresponds to the pores and a densification of 85% was calculated by the imaging method. Secondary electron images show a rounded grain appearance with a prominent grain boundary with a size in the order of 1 µm.

**Figure 6:**
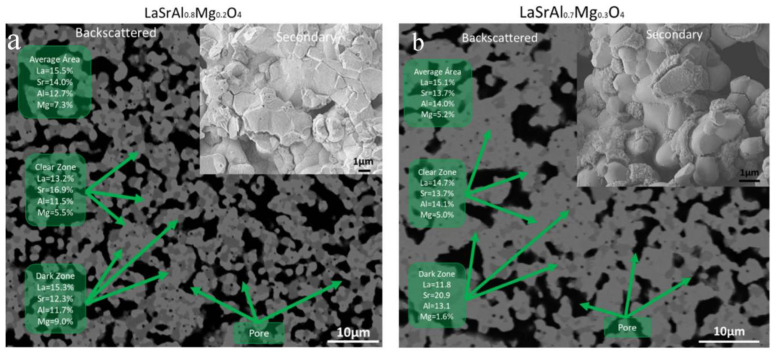
Scanning electron microscopy images of K_2_NiF_4_-type perovskites with an LaSrAl_1−x_Mg_x_O_4−δ_ (x = 0.2–0.3) composition after sintering. The green boxes present the atomic percentage data obtained by X-ray dispersive spectroscopy (EDS). (**a**) Backscattered electron and (**b**) secondary electron micrographs are presented.

Figure 6a shows the micrograph of sample x = 0.2. The backscattered electron image shows a large segregation of the La/Sr ratio with values of 1.24 and 0.78 for the dark gray and light gray areas, respectively. The segregation has an effect on the Mg concentration, which reaches 9% in the dark gray zones. The black region reveals a large porosity of the sintered pellets calculated by image analysis on the order of 70%. The secondary electron image also shows a good connectivity between the grains (a diffuse grain boundary). The size of the grains is of the order of 1 um in size.

Figure 6b shows the micrographs of sample x = 0.3. The backscattered electron image shows segregation in the La/Sr composition with values of 0.56 and 1.07 for the dark gray and light gray zones, respectively. It is observed that the excessive segregation of Sr shows an ejection of Mg atoms, decreasing their concentration considerably. The secondary electron image shows a higher densification of the order of 80%. The densification degree is, in general, low for an applicability as an electrolyte, so the sintering methods should be improved, e.g., with ultrafast sintering techniques.

### 3.4. Electric Behavior

**Figure 7:**
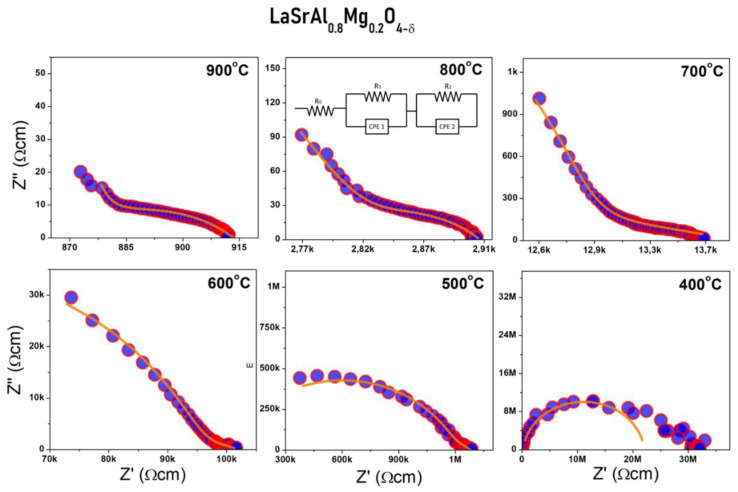
Impedance arcs at different temperatures of the sample x = 0.2. The spectra are shown together with the fit obtained by the equivalent circuit model, which is observed in the 800 °C temperature graph.

The sintered powders were studied by electrochemical impedance spectroscopy at high temperature. This characterization allows for the direct measurement of the ohmic resistance of the electrolyte at different temperatures to obtain the total electrical conductivity of the material. Figure 7 illustrates the impedance arcs in the Nyquist notation for a selected sample with x = 0.2 at different temperatures. Equivalent circuit models were used to fit the data. A model consisting of a resistor together with two CPE//R in series was used. The first two resistors correspond to the intergranular and grain-edge resistance (R_ig_ + R_gb_) and the third one corresponds to the electrode reaction. The selection of the processes involved is given by the capacitance of the CPE constant phase elements used in the model; effectively, the process will be intergranular if the capacitance is of the order of ~pF, it will be grain-edge if the capacitance is of the order of ~nF, and it will be an electrode reaction if the capacitance is of the order of ~uF.

**Figure 8:**
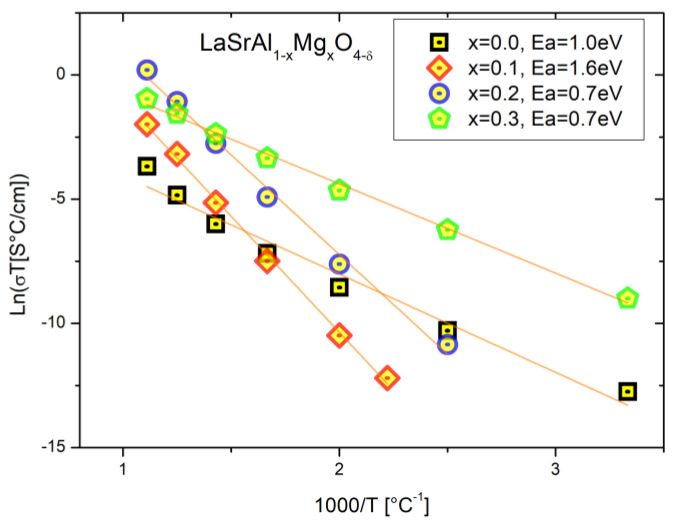
Modified Arrhenius type graph (Ln(σT) vs 1000 T^−1^). The data for the perovskites K_2_NiF_4_-type of composition LaSrAl_1−x_Mg_x_O_4−δ_ (x = 0.0–0.3) are presented, as well as the respective activation energies.

The total conductivities were plotted versus the reciprocal temperature and are presented in a modified Arrhenius-type format in Figure 8. The slope of the curve yields the activation energy required for the anion hopping from one crystallographic site to another. It is observed that for the standard sample x = 0.0, the activation energy is 1 eV with conductivities of 0.3 μS/cm (900 °C), which is consistent for this type of material with intrinsic defects. For the sample x = 0.1, the activation energy is 1.6 eV with conductivities of 0.1 mS/cm. This drives an increase of almost three orders of magnitude with the addition of Mg. For the sample x = 0.2, there is an activation energy of 1.5 eV with a conductivity of 1.4 mS/cm, that is, four orders of magnitude higher than the parent sample. This fact is consistent with the SEM-EDS data that revealed that LaSrAl_0.8_Mg_0.2_O_4_−_δ_ has the highest Mg content and shows the best intergrain connection. For x = 0.3, the activation energies are close to 1 eV, which may be due to the relative increase in electronic conductivity at medium and low temperatures. This is reflected by a decrease in the slope of the line. This effect can be due to two possible causes: i) the relatively high degree of impurities in the synthesis resulted in the nucleation and growth of second phases that could be introducing electronic currents through the grain boundary, and ii) the synthesis could have been contaminated with tungsten from mill bins and segregate at the grain boundary and introduce fugue currents.

## 4. Conclusions

In this work, the feasibility of synthesizing LaSrAl_1−x_Mg_x_O_4−δ_ layered perovskites through mechanical synthesis was studied. This is an environmentally friendly alternative versus chemical synthesis, involving the use of less polluting reactants. Indeed, LaSrAl_0.9_Mg_0.1_O_4−δ_ was obtained with a degree of purity of 99%. It was shown that the Mg incorporation improves the crystallinity of the samples prior to annealing. Electron diffraction and TEM data indicated that the obtained particles have a size of the order of 10 nm with a nanometer-scale crystalline order, combined with amorphous regions of incomplete transformation. After annealing at 1100 °C and sintering at 1400 °C for 10 h, there is a full growth of the parent phase LaSrAl_1_−_x_Mg_x_O_4_−_δ_, which, for x = 0.1, is completely pure. For x = 0.2 and x = 0.3, there is the growth of minor impurities identified as La_2_SrAl_2_O_7_, LaSrAl_3_O_7_, La_2_O_3_, and SrO, which can have a counterproductive effect on the ionic conductivity. Differential scanning calorimetry studies show that the energy needed to obtain the phase in previously milled samples is small compared to the chemical synthesis because phase formation occurs in mechanical synthesis and annealing translates only as grain growth. On the other hand, it was seen by SEM and EDS that for x = 0.2 and x = 0.3, there is a local segregation of La and Sr, which may have an effect on the nucleation of these impurities, either due to stacking disorders (La_2_SrAl_2_O_7_) or simply the stability of the impure phases against Mg-doped layered perovskites of the K_2_NiF_4_ type. The latter may have too many lattice strains due to the difference in the Al^3+^–Mg^2+^ ionic radii. The porosities of the materials were high, of the order of 30%, which must be minimized so that they have applicability as solid electrolytes. The ionic conductivities were also considerably improved with the incorporation of Mg in the synthesis, obtaining an increase of up to four orders of magnitude. Finally, a conductivity of 0.1 mS/cm and 1.6 mS/cm at 900 °C was obtained for x = 0.1 and x = 0.2, respectively, versus the undoped sample with a conductivity of 0.3 μS/cm. For x = 0.3, the segregation of impurities and possible tungsten contamination from the ball mill containers could be introducing electronic currents that are translated into a decrease in the activation energies. It is imperative to continue working through different synthesis and sintering strategies to improve the densification of the pellets so as to avoid the segregation of heavy ions. (The Supplementary Material shows the original EDS spectra. In case the reader wants to check the results shown in the micrographs).

## Figures and Tables

**Table 1:** Structural parameters determined by Rietveld refinement from laboratory XRD data.

LaSrAl_1−x_Mg_x_O_4_	x = 0.0	x = 0.1	x = 0.2	x = 0.3
a[Å]	3.7606 (7)	3.76819 (11)	3.7770 (2)	3.7897 (3)
c[Å]	12.678 (3)	12.6985 (4)	12.7174 (8)	12.727 (1)
V[Å^3^]	179.29 (6)	180.31 (1)	181.43 (2)	182.78 (2)
La/Sr (0 0 z)				
z	0.3569 (3)	0.35885 (9)	0.35901 (14)	0.3599 (2)
f_occ_	1.0	1.0	1.0	1.0
Al/Mg (½ ½ ½)				
f_occ_	1.0/0.0	0.9/0.1	0.8/0.2	0.7/0.3
O1 (½ 0 ½)				
B [Å^2^]	-	2.3 (5)	1.4 (7)	1.7 (9)
f_occ_	2.0	2.00 (0)	2.00(0)	2.00 (0)
O2 (0 0 z)				
z	0.163 (2)	0.1642 (5)	0.1642 (8)	0.1662 (1)
B [Å^2^]	-	1.7 (4)	0.9 (5)	0.3 (7)
f_occ_	2.0	2.00 (0)	2.00 (0)	2.00 (0)
Reliability	parameters			
χ^2^	2.77	1.57	2.43	6.37
R_p_ (%)	2.57	4.73	6.20	7.43
R_wp_ (%)	3.49	5.95	8.40	12.2
R_Bragg_ (%)	5.77	2.78	4.84	4.53

**Table 2:** Main distances and bond angles for the powder samples.

Distance [Å]	LaSrAlO_4_	LaSrAl_0.9_Mg_0.1_O_4_	LaSrAl_0.8_Mg_0.2_O_4_	LaSrAl_0.7_Mg_0.3_O_4_
La/Sr-O1(x4)	2.613 (3)	2.6005 (8)	2.6041 (12)	2.6019 (17)
La/Sr-O2	2.46 (2)	2.472 (6)	2.477 (10)	2.464 (13)
La/Sr-O2(x4)	2.671 (2)	2.6805 (7)	2.6870 (11)	2.7004 (16)
$\langle La/Sr-O\rangle$	2.623 (2)	2.622 (1)	2.627 (2)	2.630 (3)
Al/Mg-O1(x4)	1.8803 (4)	1.88409 (5)	1.88853 (9)	1.89485 (10)
Al/Mg-O2(x2)	2.07 (2)	2.085 (6)	2.088 (10)	2.117 (13)
$\langle Al/Mg-O\rangle$	1.944 (7)	1.951 (2)	1.955 (4)	1.969 (22)
O1-O1(x4)	2.6591 (4)	2.66451 (5)	2.67078 (9)	2.67972 (10)
O2-O1(x4)	2.793 (17)	2.810 (5)	2.816 (8)	2.841 (9)
O2-O2(x4)	3.46 (2)	3.442 (6)	3.449 (9)	3.423 (11)
Al/Mg-O1-Al/Mg	180.0	180.0	180.0	180.0
Al/Mg-O2-Al/Mg	180.0	180.0	180.0	180.0

## Data Availability

Data are contained within the article.

## Supplementary Materials

The following are available online at https://www.mdpi.com/article/10.3390/ma16247564/s1, Pages 1–2: LaSrAlO_4_, Pages 3–6: LaSrAl_0.9_Mg_0.1_O_4_, Pages 7–13: LaSrAl_0.8_Mg_0.2_O_4_, Pages 14–17: LaSrAl_0.7_Mg_0.3_O_4._
