# Supplementary material for: Structural and Electrical Characterization of LaSrAl1−xMgxO4−δ Layered Perovskites Obtained by Mechanical Synthesis
Source: Materials (Basel). 2023 Dec 8;16(24):7564. doi: 10.3390/ma16247564 (PMC10745031; doi:10.3390/ma16247564)
Supplement: Supplementary file 1 [file materials-16-07564-s001.zip › materials-2653778-supplementary.pdf]

Carlos Marinio

Author: Paula

Creation: 09/23/2019 2:33:37 PM

Sample Name: P-SM-Al

Area 1

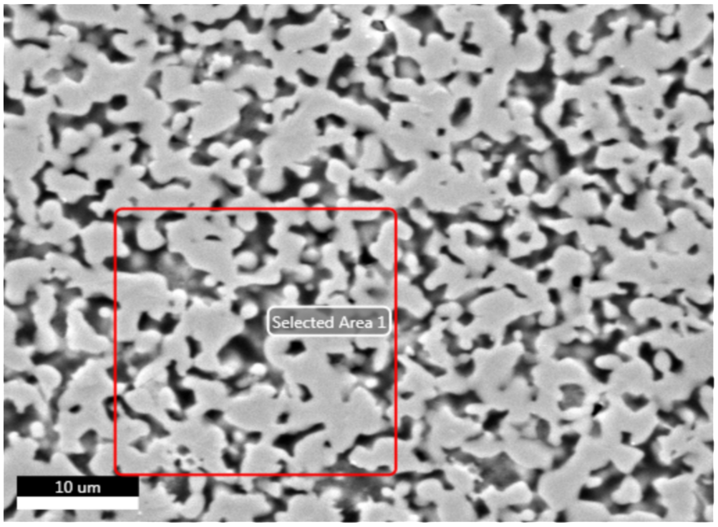

Notes:

Selected Area 1

kV: 20	Mag: 5007	Takeoff: 35.2	Live Time(s): 30	Amp Time(μs): 3.84	Resolution:(eV) 123.8

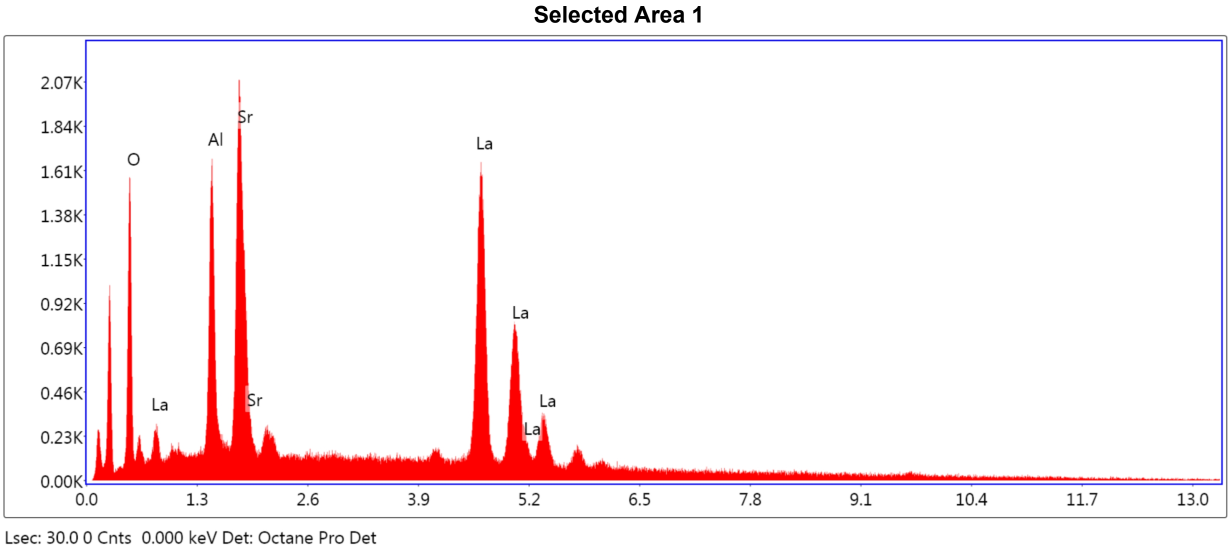

eZAF Smart Quant Results

Element	Weight %	Atomic %	Net Int.	Error %	Kratio	Z	R	A	F
O K	17.0	50.6	507.4	9.3	0.0594	1.3303	0.8174	0.2621	1.0000
AlK	11.4	20.0	754.4	8.4	0.0506	1.1971	0.8671	0.3689	1.0063
SrL	24.1	13.1	932.8	5.8	0.1391	0.9414	1.0332	0.6068	1.0101
LaL	47.5	16.2	1213.1	3.0	0.4242	0.8514	1.1183	1.0173	1.0316

Carlos Marinio

Author: Paula
Creation: 09/23/2019 2:11:05 PM

Sample Name: New Sample

Area 1

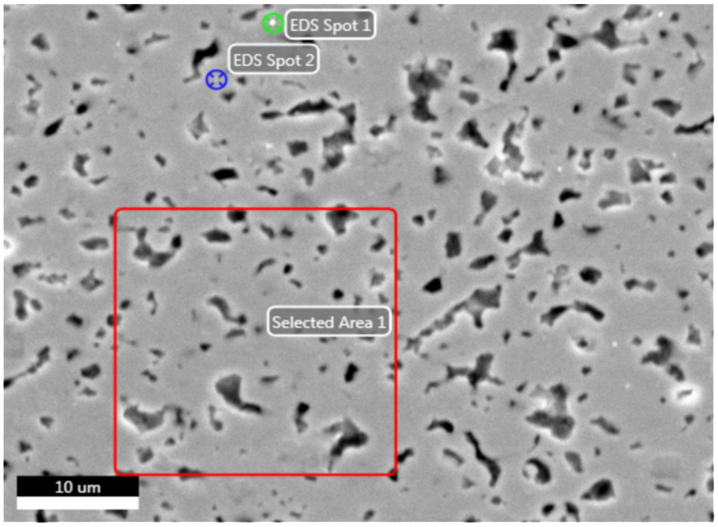

Notes:

Selected Area 1

kV: 20	Mag: 5007	Takeoff: 35.2	Live Time(s): 30	Amp Time(μs): 3.84	Resolution:(eV) 123.8

Selected Area 1

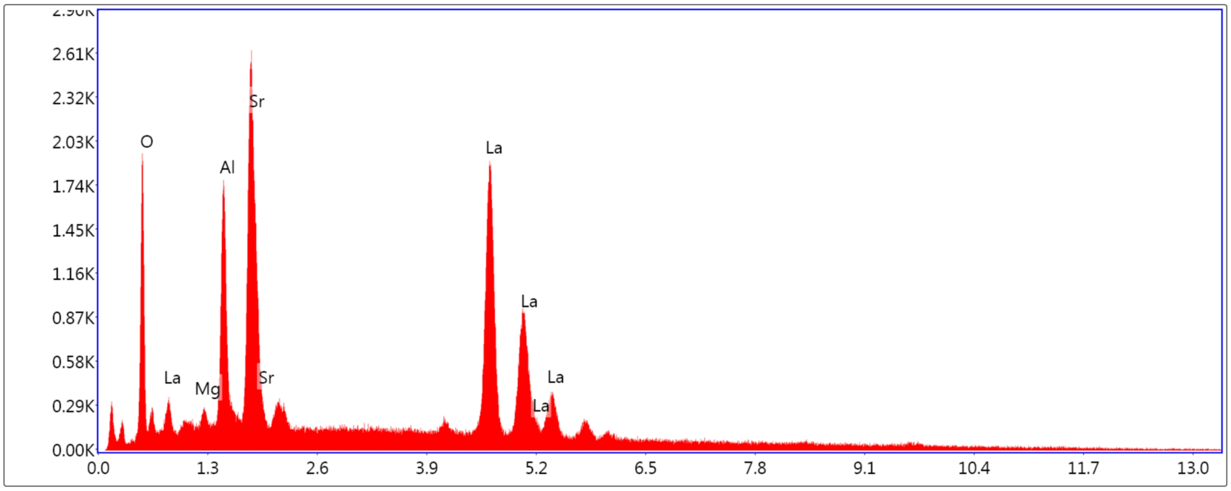

Lsec: 30.0 0 Cnts 0.000 keV Det: Octane Pro Det

<u>eZAF Smart Quant Results</u>

Element	Weight %	Atomic %	Net Int.	Error %	Kratio	Z	R	A	F
O K	17.4	50.8	619.9	9.2	0.0593	1.3250	0.8199	0.2577	1.0000
MgK	1.5	2.8	93.2	12.5	0.0051	1.2354	0.8604	0.2786	1.0047
AlK	10.1	17.4	816.7	8.3	0.0447	1.1922	0.8696	0.3704	1.0064
SrL	25.5	13.6	1222.4	5.6	0.1488	0.9376	1.0361	0.6162	1.0098
LaL	45.6	15.3	1420.3	2.7	0.4057	0.8478	1.1209	1.0173	1.0319

EDS Spot 1

kV: 20	Mag: 5007	Takeoff: 35.2	Live Time(s): 30	Amp Time(μs): 3.84	Resolution:(eV) 123.8

EDS Spot 1

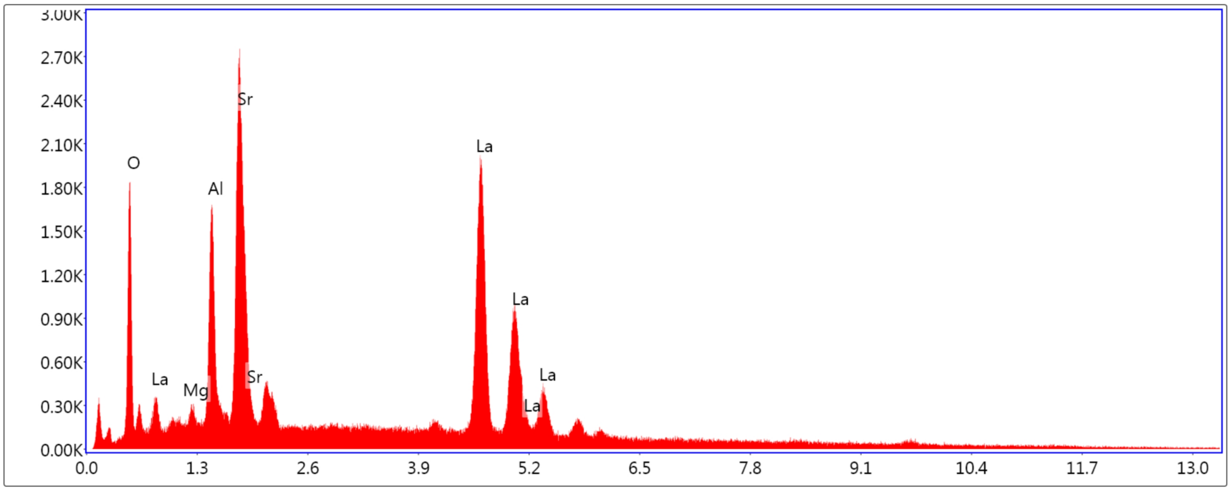

eZAF Smart Quant Results

Element	Weight %	Atomic %	Net Int.	Error %	Kratio	Z	R	A	F
O K	16.7	50.0	610.5	9.2	0.0573	1.3316	0.8166	0.2577	1.0000
MgK	1.6	3.3	104.9	12.4	0.0056	1.2416	0.8570	0.2744	1.0046
AlK	9.4	16.7	767.8	8.4	0.0412	1.1982	0.8663	0.3646	1.0064
SrL	25.4	13.9	1242.1	5.6	0.1483	0.9423	1.0322	0.6126	1.0099
LaL	46.8	16.2	1493.2	2.7	0.4184	0.8523	1.1174	1.0162	1.0317

EDS Spot 2

kV: 20	Mag: 5007	Takeoff: 35.2	Live Time(s): 8	Amp Time(μs): 3.84	Resolution:(eV) 123.8

EDS Spot 2

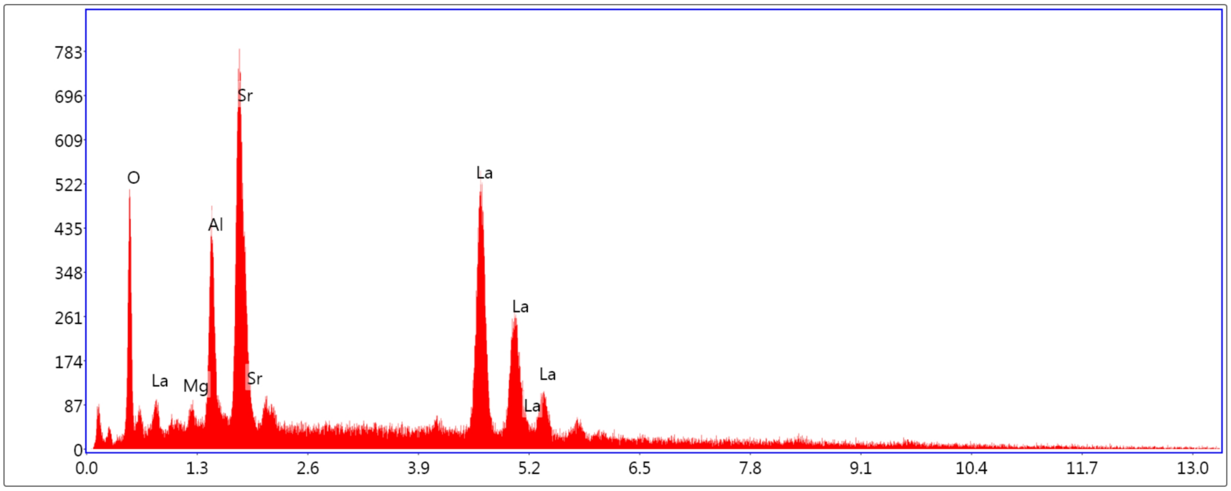

Lsec: 8.0 0 Cnts 0.000 keV Det: Octane Pro Det

eZAF Smart Quant Results

Element	Weight %	Atomic %	Net Int.	Error %	Kratio	Z	R	A	F
O K	17.0	50.6	609.3	10.2	0.0569	1.3297	0.8176	0.2521	1.0000
MgK	1.6	3.2	105.0	17.2	0.0056	1.2398	0.8580	0.2787	1.0047
AlK	9.1	16.0	756.6	9.4	0.0404	1.1965	0.8673	0.3699	1.0066
SrL	27.0	14.7	1338.9	6.5	0.1590	0.9409	1.0334	0.6207	1.0096
LaL	45.4	15.6	1452.5	4.9	0.4047	0.8510	1.1185	1.0154	1.0319

Carlos Marinio

Author: Paula
Creation: 09/24/2019 10:07:16 AM

Sample Name: Mg-02-SM

Area 1

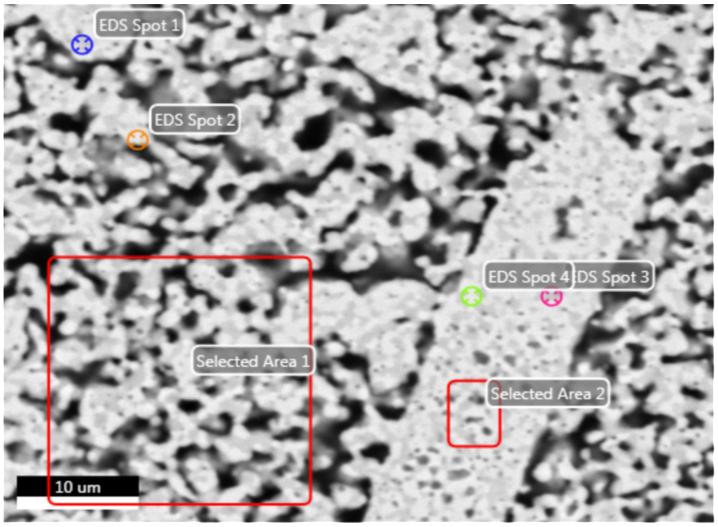

Notes:

Selected Area 1

kV: 20	Mag: 5007	Takeoff: 35.2	Live Time(s): 30	Amp Time(μs): 3.84	Resolution:(eV) 123.8

Selected Area 1

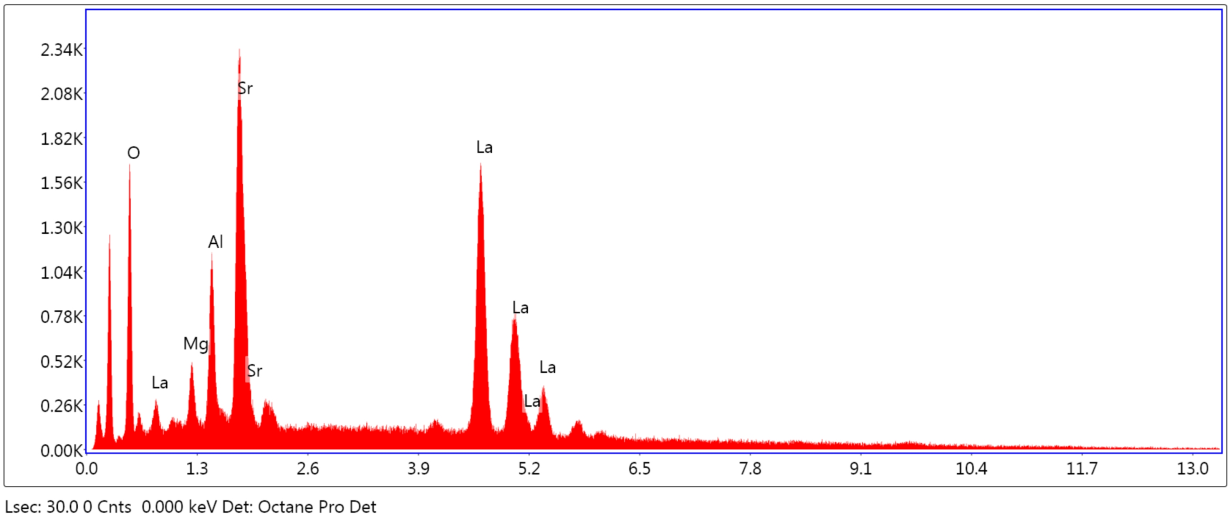

eZAF Smart Quant Results

Element	Weight %	Atomic %	Net Int.	Error %	Kratio	Z	R	A	F
O K	17.1	50.4	527.9	9.3	0.0584	1.3265	0.8186	0.2571	1.0000
MgK	3.8	7.3	206.1	10.4	0.0131	1.2368	0.8591	0.2777	1.0044
AlK	7.3	12.7	496.7	8.8	0.0314	1.1936	0.8683	0.3597	1.0065
SrL	26.1	14.0	1086.0	5.7	0.1529	0.9387	1.0346	0.6189	1.0098
LaL	45.8	15.5	1234.8	2.9	0.4075	0.8489	1.1195	1.0169	1.0319

Selected Area 2

kV: 20	Mag: 5007	Takeoff: 35.2	Live Time(s): 30	Amp Time(μs): 3.84	Resolution:(eV) 123.8

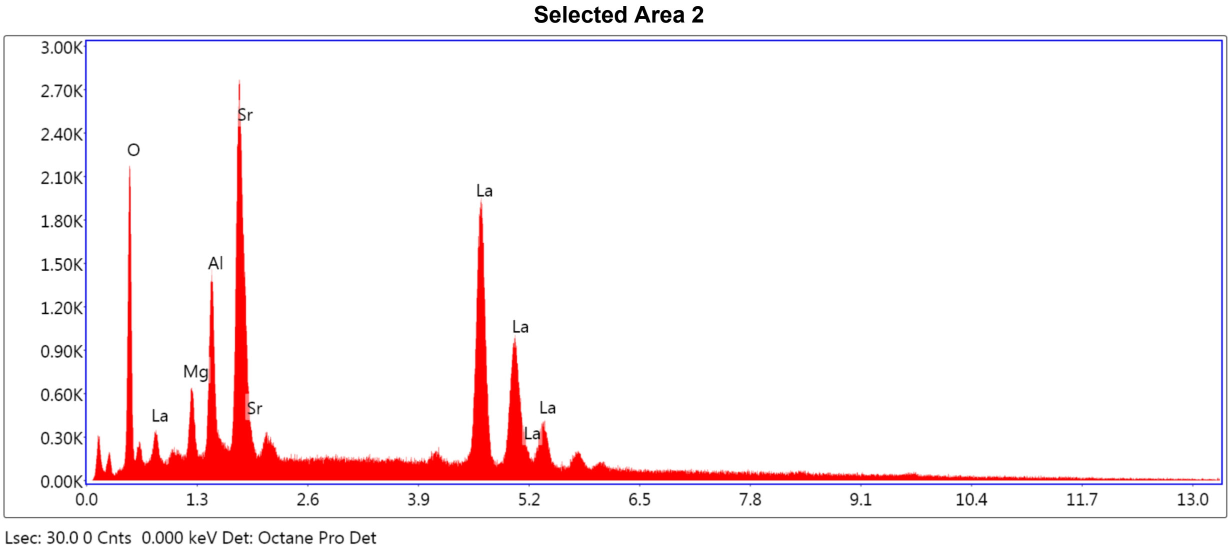

eZAF Smart Quant Results

Element	Weight %	Atomic %	Net Int.	Error %	Kratio	Z	R	A	F
O K	18.6	52.4	709.9	9.1	0.0640	1.3156	0.8237	0.2623	1.0000
MgK	3.9	7.2	258.8	10.1	0.0134	1.2267	0.8642	0.2814	1.0045
AlK	7.8	13.0	651.7	8.5	0.0336	1.1838	0.8735	0.3639	1.0065
SrL	25.2	13.0	1284.8	5.6	0.1475	0.9310	1.0406	0.6222	1.0098
LaL	44.6	14.5	1468.2	2.7	0.3951	0.8415	1.1249	1.0197	1.0320

EDS Spot 1

kV: 20	Mag: 5007	Takeoff: 35.2	Live Time(s): 30	Amp Time(μs): 3.84	Resolution:(eV) 123.8

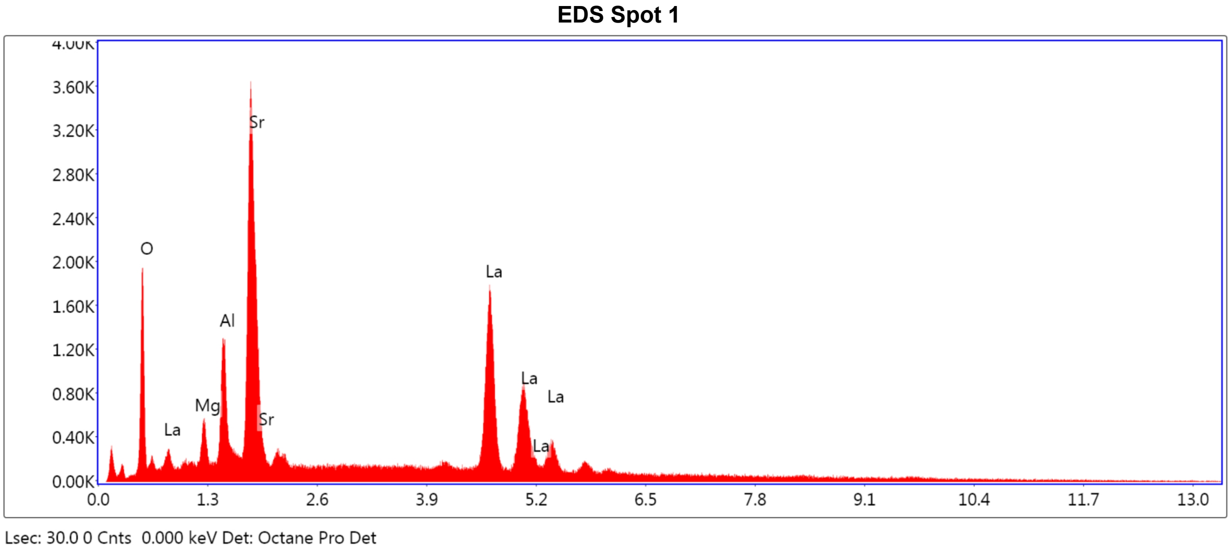

eZAF Smart Quant Results

Element	Weight %	Atomic %	Net Int.	Error %	Kratio	Z	R	A	F
O K	18.4	52.9	626.1	9.4	0.0571	1.3182	0.8231	0.2355	1.0000
MgK	2.9	5.5	204.8	10.6	0.0107	1.2291	0.8636	0.2965	1.0048
AlK	6.8	11.5	597.5	8.4	0.0311	1.1861	0.8729	0.3855	1.0072
SrL	32.2	16.9	1709.6	5.1	0.1982	0.9327	1.0399	0.6539	1.0087
LaL	39.7	13.2	1290.3	2.8	0.3507	0.8431	1.1243	1.0140	1.0329

EDS Spot 2

kV: 20	Mag: 5007	Takeoff: 35.2	Live Time(s): 30	Amp Time(μs): 3.84	Resolution:(eV) 123.8

EDS Spot 2

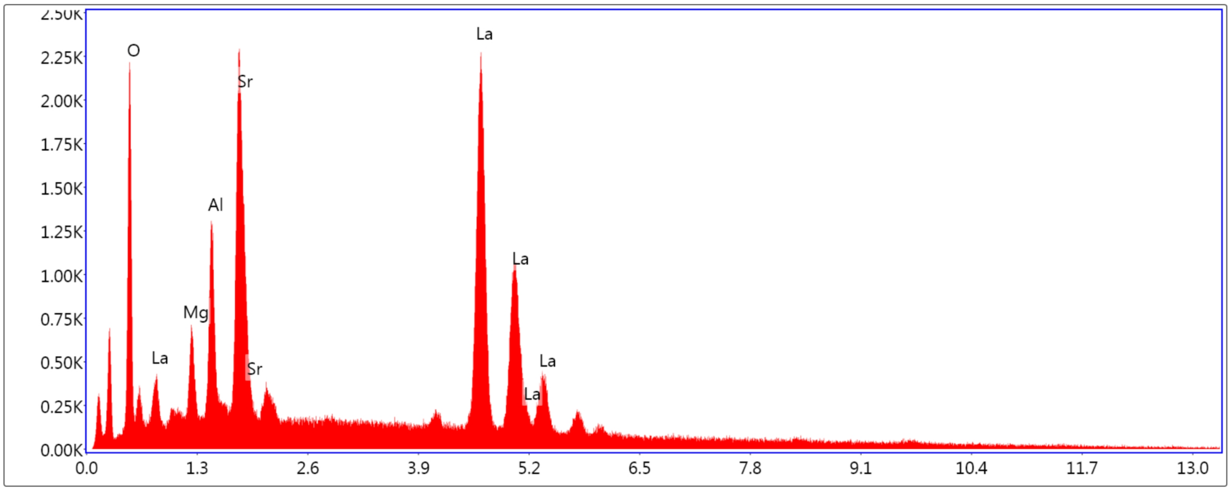

Lsec: 30.0 0 Cnts 0.000 keV Det: Octane Pro Det

eZAF Smart Quant Results

Element	Weight %	Atomic %	Net Int.	Error %	Kratio	Z	R	A	F
O K	17.5	50.9	729.5	8.9	0.0652	1.3249	0.8186	0.2806	1.0000
MgK	4.6	8.8	298.8	10.2	0.0153	1.2354	0.8591	0.2664	1.0042
AlK	7.3	12.6	591.2	8.8	0.0302	1.1922	0.8683	0.3434	1.0061
SrL	20.9	11.1	1042.9	5.9	0.1186	0.9376	1.0346	0.5985	1.0107
LaL	49.6	16.6	1661.6	2.7	0.4429	0.8480	1.1196	1.0213	1.0314

EDS Spot 3

kV: 20	Mag: 5007	Takeoff: 35.2	Live Time(s): 30	Amp Time(μs): 3.84	Resolution:(eV) 123.8

EDS Spot 3

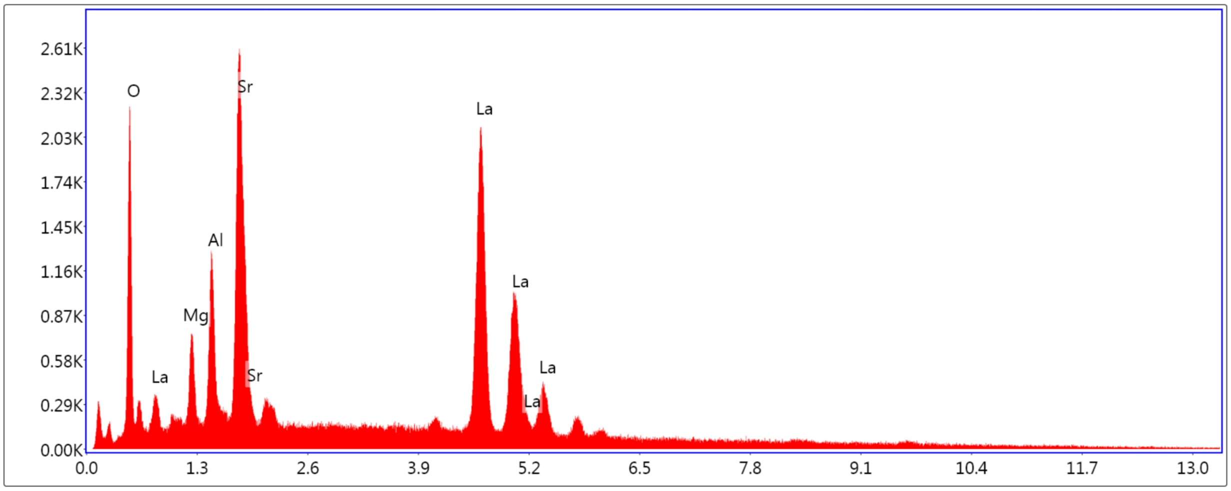

Lsec: 30.0 0 Cnts 0.000 keV Det: Octane Pro Det

eZAF Smart Quant Results

Element	Weight %	Atomic %	Net Int.	Error %	Kratio	Z	R	A	F
O K	18.1	51.6	720.6	9.0	0.0642	1.3194	0.8215	0.2694	1.0000
MgK	4.8	9.0	320.6	10.1	0.0164	1.2303	0.8620	0.2754	1.0043
AlK	6.9	11.7	574.0	8.7	0.0292	1.1872	0.8713	0.3531	1.0063
SrL	23.6	12.3	1203.0	5.7	0.1364	0.9337	1.0380	0.6131	1.0101
LaL	46.6	15.3	1558.1	2.7	0.4141	0.8442	1.1226	1.0203	1.0318

EDS Spot 4

kV: 20	Mag: 5007	Takeoff: 35.2	Live Time(s): 30	Amp Time(μs): 3.84	Resolution:(eV) 123.8

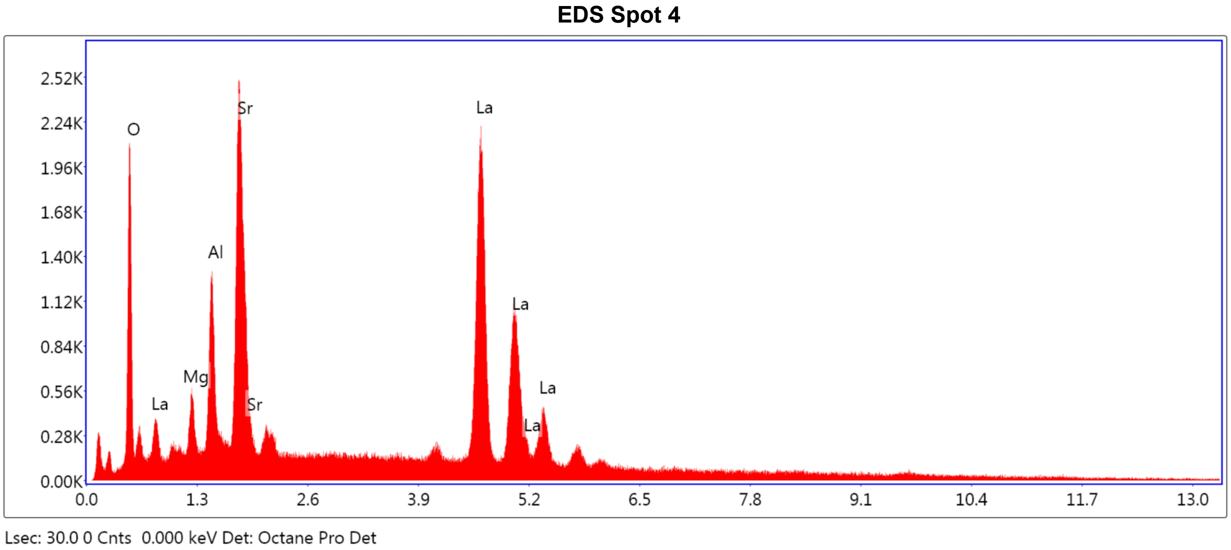

eZAF Smart Quant Results

Element	Weight %	Atomic %	Net Int.	Error %	Kratio	Z	R	A	F
O K	17.4	51.4	700.9	9.0	0.0625	1.3300	0.8164	0.2706	1.0000
MgK	3.5	6.8	228.0	10.4	0.0117	1.2401	0.8568	0.2674	1.0043
AlK	7.2	12.7	596.0	8.7	0.0304	1.1968	0.8661	0.3489	1.0063
SrL	23.0	12.4	1169.2	5.7	0.1326	0.9412	1.0319	0.6056	1.0104
LaL	48.9	16.7	1644.4	2.6	0.4373	0.8514	1.1172	1.0188	1.0315

EDAX TEAM

Carlos Marinio

Author: Paula
Creation: 09/24/2019 11:03:02 AM

Sample Name: Mg-03-SM

Area 1

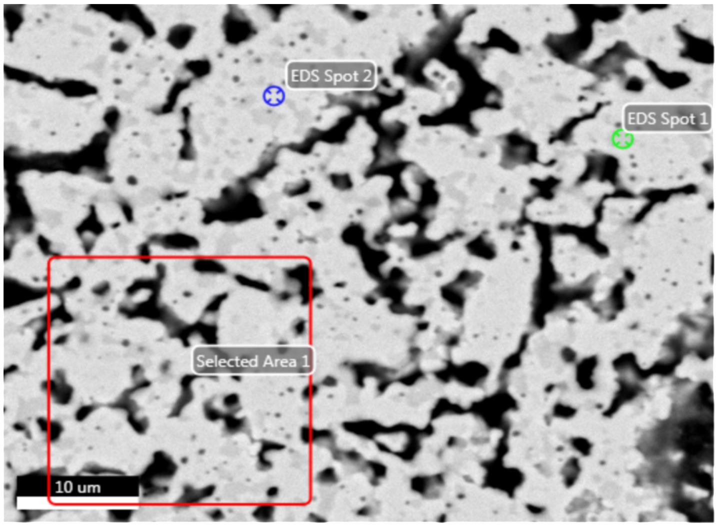

Notes:

Selected Area 1

kV: 20	Mag: 5007	Takeoff: 35.2	Live Time(s): 30	Amp Time(µs): 3.84	Resolution:(eV) 123.8

Selected Area 1

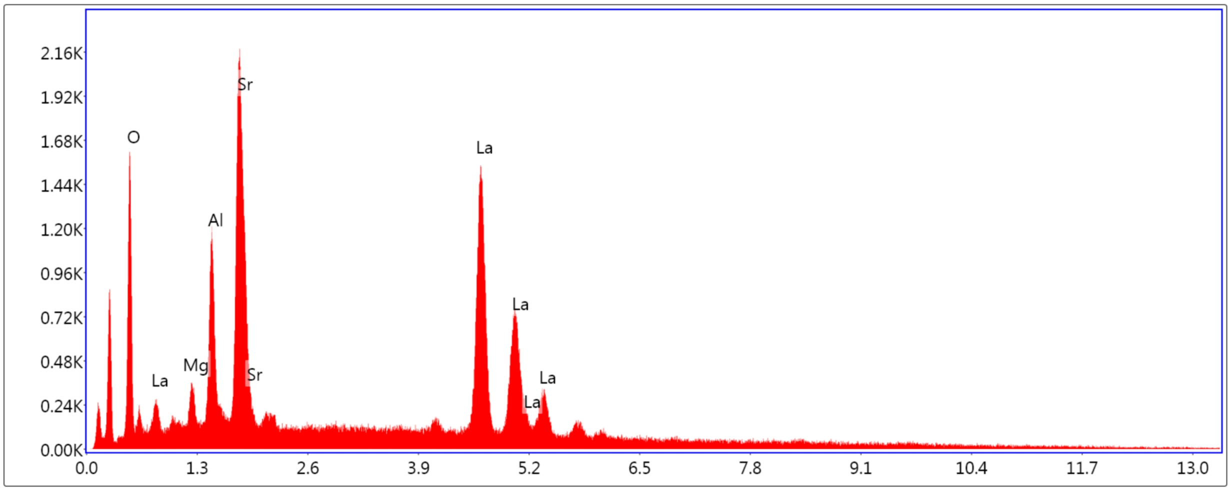

Lsec: 30.0 0 Cnts 0.000 keV Det: Octane Pro Det

eZAF Smart Quant Results

Element	Weight %	Atomic %	Net Int.	Error %	Kratio	Z	R	A	F
O K	18.0	52.1	522.5	9.3	0.0615	1.3225	0.8206	0.2582	1.0000
MgK	2.7	5.2	140.0	11.2	0.0094	1.2331	0.8611	0.2789	1.0045
AlK	8.1	14.0	530.2	8.6	0.0357	1.1900	0.8703	0.3656	1.0065
SrL	25.9	13.7	1017.5	5.7	0.1523	0.9358	1.0369	0.6215	1.0097
LaL	45.2	15.1	1144.8	2.9	0.4017	0.8462	1.1216	1.0178	1.0320

EDS Spot 1

kV: 20	Mag: 5007	Takeoff: 35.2	Live Time(s): 30	Amp Time(μs): 3.84	Resolution:(eV) 123.8

EDS Spot 1

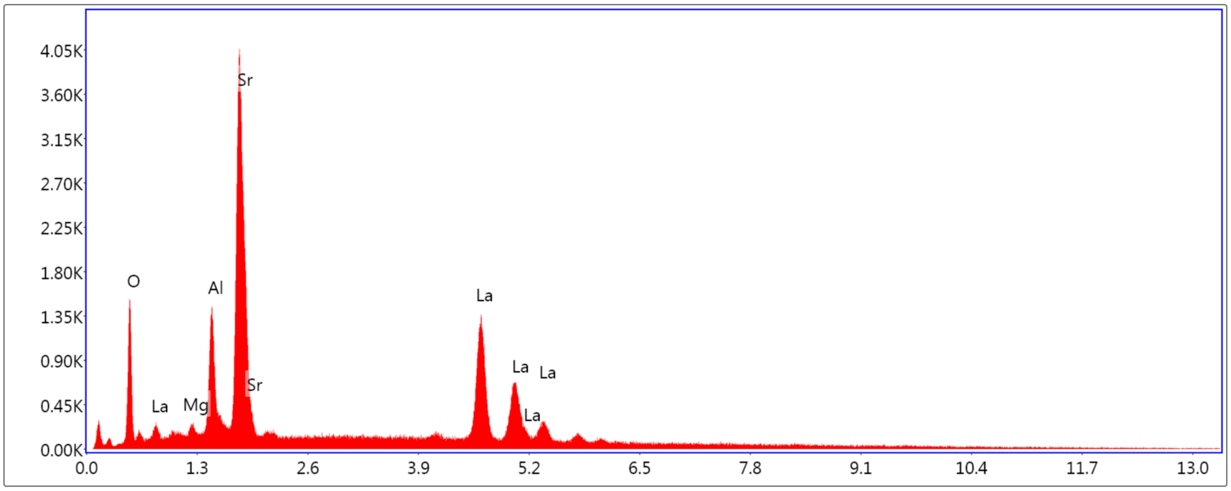

Lsec: 30.0 0 Cnts 0.000 keV Det: Octane Pro Det

eZAF Smart Quant Results

Element	Weight %	Atomic %	Net Int.	Error %	Kratio	Z	R	A	F
O K	17.9	52.5	479.2	9.8	0.0502	1.3214	0.8227	0.2128	1.0000
MgK	0.8	1.6	54.8	15.6	0.0033	1.2320	0.8632	0.3133	1.0054
AlK	7.5	13.1	624.8	8.0	0.0374	1.1889	0.8725	0.4153	1.0080
SrL	38.9	20.9	1879.5	4.8	0.2506	0.9349	1.0394	0.6839	1.0079
LaL	34.9	11.8	983.1	3.4	0.3073	0.8451	1.1239	1.0081	1.0341

EDS Spot 2

kV: 20	Mag: 5007	Takeoff: 35.2	Live Time(s): 30	Amp Time(µs): 3.84	Resolution:(eV) 123.8

EDS Spot 2

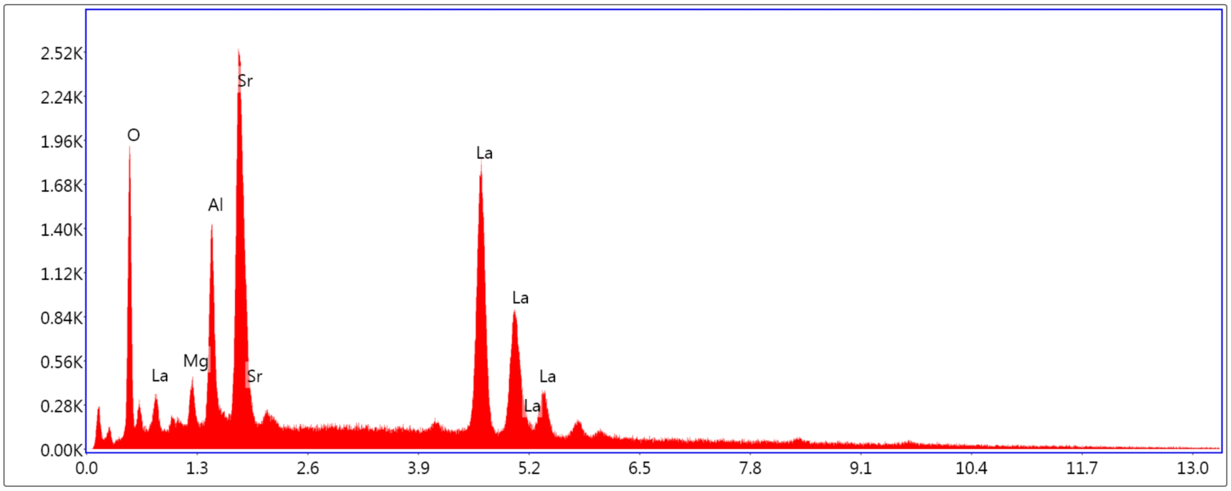

Lsec: 30.0 0 Cnts 0.000 keV Det: Octane Pro Det

eZAF Smart Quant Results

Element	Weight %	Atomic %	Net Int.	Error %	Kratio	Z	R	A	F
O K	18.3	52.5	621.7	9.2	0.0623	1.3197	0.8219	0.2574	1.0000
MgK	2.7	5.0	161.9	11.0	0.0093	1.2306	0.8624	0.2811	1.0046
AlK	8.3	14.1	636.3	8.5	0.0364	1.1875	0.8717	0.3685	1.0065
SrL	26.2	13.7	1210.6	5.6	0.1542	0.9339	1.0385	0.6244	1.0097
LaL	44.5	14.7	1322.0	2.8	0.3950	0.8443	1.1231	1.0181	1.0320
